# A comprehensive quantitative phosphoproteome analysis of rice in response to bacterial blight

**DOI:** 10.1186/s12870-015-0541-2

**Published:** 2015-06-26

**Authors:** Yuxuan Hou, Jiehua Qiu, Xiaohong Tong, Xiangjin Wei, Babi R. Nallamilli, Weihuai Wu, Shiwen Huang, Jian Zhang

**Affiliations:** China National Rice Research Institute, Hangzhou, 311400 China; Department of Human Genetics, Emory University School of Medicine, Atlanta, GA 30322 U.S.A.; Hainan Key Laboratory for Monitoring and Control of Tropical Agricultural Pests, Environment and Plant Protection Institute, Chinese Academy of Tropical Agricultural Sciences, Haikou, Hainan 571101 China

**Keywords:** Rice (*Oryza sativa* L.), Phosphoproteome, Bacterial blight, Post-translational modification

## Abstract

**Background:**

Rice is a major crop worldwide. Bacterial blight (BB) caused by *Xanthomonas oryzae* pv. *oryzae* (*Xoo*) has become one of the most devastating diseases for rice. It has been clear that phosphorylation plays essential roles in plant disease resistance. However, the role of phosphorylation is poorly understood in rice-*Xoo* system. Here, we report the first study on large scale enrichment of phosphopeptides and identification of phosphosites in rice before and 24 h after *Xoo* infection.

**Results:**

We have successfully identified 2367 and 2223 phosphosites on 1334 and 1297 representative proteins in 0 h and 24 h after *Xoo* infection, respectively. A total of 762 differentially phosphorylated proteins, including transcription factors, kinases, epi-genetic controlling factors and many well-known disease resistant proteins, are identified after *Xoo* infection suggesting that they may be functionally relevant to *Xoo* resistance. In particular, we found that phosphorylation/dephosphorylation might be a key switch turning on/off many epi-genetic controlling factors, including HDT701, in response to *Xoo* infection, suggesting that phosphorylation switch overriding the epi-genetic regulation may be a very universal model in the plant disease resistance pathway.

**Conclusions:**

The phosphosites identified in this study would be a big complementation to our current knowledge in the phosphorylation status and sites of rice proteins. This research represents a substantial advance in understanding the rice phosphoproteome as well as the mechanism of rice bacterial blight resistance.

**Electronic supplementary material:**

The online version of this article (doi:10.1186/s12870-015-0541-2) contains supplementary material, which is available to authorized users.

## Background

During the whole life cycle, plants are continuously threatened by different pathogens including bacteria, fungi and virus. To survive under the pathogen invasion, plants build up their primary defense by using a structural barrier like the cell wall or cuticle, which is a non-host resistance but also can be easily conquered by pathogens. After the collapse of the primary defense, the secondary defense of plants, a more pronounced defense than the primary one, could be triggered by effector proteins that are secreted by plant pathogens. Therefore, the recognition of effector proteins and signal transduction in the second defense are of great importance in the plant-pathogen interaction study.

Recent studies have revealed that besides the quantity of protein synthesis, post-translational modification (PTM) of the pre-existing signaling proteins is also critical in the signal transduction cascade to ensure that plants respond to the pathogen invasion in a prompt manner [1]. So far, among the PTMs reported in defense signaling, phosphorylation is the most common and intensively studied one. Phosphorylation is a reversible, covalent modification usually occurring on the hydroxyl group of hydroxyl amino acids like serine, threonine and tyrosine, but occasionally on hydroxyl-proline [2]. Phosphorylation and dephosphorylation on specific sites of proteins are catalyzed by kinases and phosphatases respectively to alter the protein nature and configuration and ultimately provide modified protein with new functions in enzyme activity, substrate specificity, structure stability or intracellular localization. Phosphorylation is a very abundant modification in plant and animal proteins. It was also suggested that more than one-third of all proteins are potentially phosphorylated [3] with diverse roles in different metabolic pathways and disease signaling. Therefore, the large number of phosphorylated proteins together with the transient, reversible phosphorylation patterns enables plants to own highly dynamic, complex signaling cascades in defense to the pathogen infection. Since the discovery of protein phosphorylation from parsley cells upon fungal infection in 1990, our knowledge about phosphorylation in plant-pathogen signaling pathway has been largely expanded [4]. Protein phosphorylation participated in the whole process of plant-pathogen interaction, including the signal perception, early signaling transduction as well as the immune response activation [1]. To sense the pathogen signals, an auto-phosphorylation of the receptor-like kinases (RLKs) on the kinase domain is required in *Arabidopsis*. Mutation in the phosphosites could abolish or weaken the signaling in downstream genes [5, 6]. In plants, the signals from the upstream elicitor receptors/sensors to the downstream MAPK (Mitogen-Activated Protein Kinase) substrates largely rely on the three-step MAPKKK (MAP Kinase Kinase Kinase)-MAPKK (MAP Kinase Kinase)-MAPK cascade [7]. The signals from receptor kinase could be transmitted and amplified from MAPKKK to downstream MAPKK, then to MAPK via phosphorylating certain sites of the downstream substrates on each step, and eventually convert signals generated at the receptors into cellular responses in plants. Such an MAPK signaling cascade plays vital roles in plant defense signaling.

Given the importance of protein phosphorylation in plant defense signaling, extensive studies have been carried out with tremendous progress achieved in the past decades. Nevertheless, due to the technical bottlenecks, traditional researches usually studied the kinase-substrate pairs one by one, and the phosphosites are determined through amino acid sites mutation of the substrate proteins, which makes the identification of phosphosites on proteins extremely challenging and tedious. As a result of the recent development of novel methods in phosphopeptides enrichment and mass spectrometry, high through-put identification of the phosphopeptides and phosphosites in the proteome level have become available. In 2006, a phosphoproteomic survey resulted in the detection of 6600 phosphosites on 2244 proteins in human HeLa cells [8]. Villen et al. reported the identification of 5635 non-redundant phosphosites from 2328 proteins from mouse liver [9]. Up to now, the PhosphoSitePlus website (http://www.phosphosite.org) has accumulated over 145,000 literatures describing 246,713 phosphosites of 19,717 proteins from various tissues and species [10]. According to P3DB database (Plant Protein Phosphorylation Database, http://p3db.org/), 32 independent phosphoproteome studies have generated the data of 47,923 phosphosites in 16,477 phosphoproteins from *Arabidopsis*, *Medicago*, rice and other 6 plant organisms [11].

Rice (*Oryza sativa* L.) is one of the most important food crops in the world, providing approximately 21 % of the calories for over half of the global population [12]. Bacterial blight (BB) caused by *Xanthomonas oryzae* pv. *oryzae* (*Xoo*) has become one of the most devastating diseases of rice worldwide as the yield loss can be up to 50 % or more. Meanwhile, rice-*Xoo* system provides an ideal model for studying plant-pathogen cross-talk due to the availability of genome sequences and ample genetic variations of both partners [13]. Even though large number of phosphoproteomic studies has documented more phosphosites in different plant species, the role of phosphorylation is poorly understood in plant-bacterial interactions especially in the rice-*Xoo* system. Therefore, large-scale identification of phosphoproteins and phosphosites of rice in response to *Xoo* infection is of great significance to reveal the disease signal transduction pathway, and how the pathogen surpasses rice defense that leads to rice resistance or susceptibility. Here, we report the first study on large scale enrichment of phosphopeptides and identification of phosphosites in rice before and 24 h after *Xoo* infection. We have successfully identified 2223 phosphosites on 1297 representative proteins after 24 h of *Xoo* infection. A total of 762 differentially phosphorylated proteins were identified after *Xoo* infection suggesting that they may be functionally relevant to disease resistance. Current phosphoproteomic study ultimately improved our understanding of signal transduction in rice disease resistance. To the best of our knowledge, this is the first phosphoproteomic report regarding the rice-*Xoo* interaction. The information obtained in this study would substantially advance our understanding of the signal transduction in rice disease resistance.

## Results

### Phosphorylation dynamics of rice variety IRBB5 in response to *Xoo* infection

**Fig. 1:**
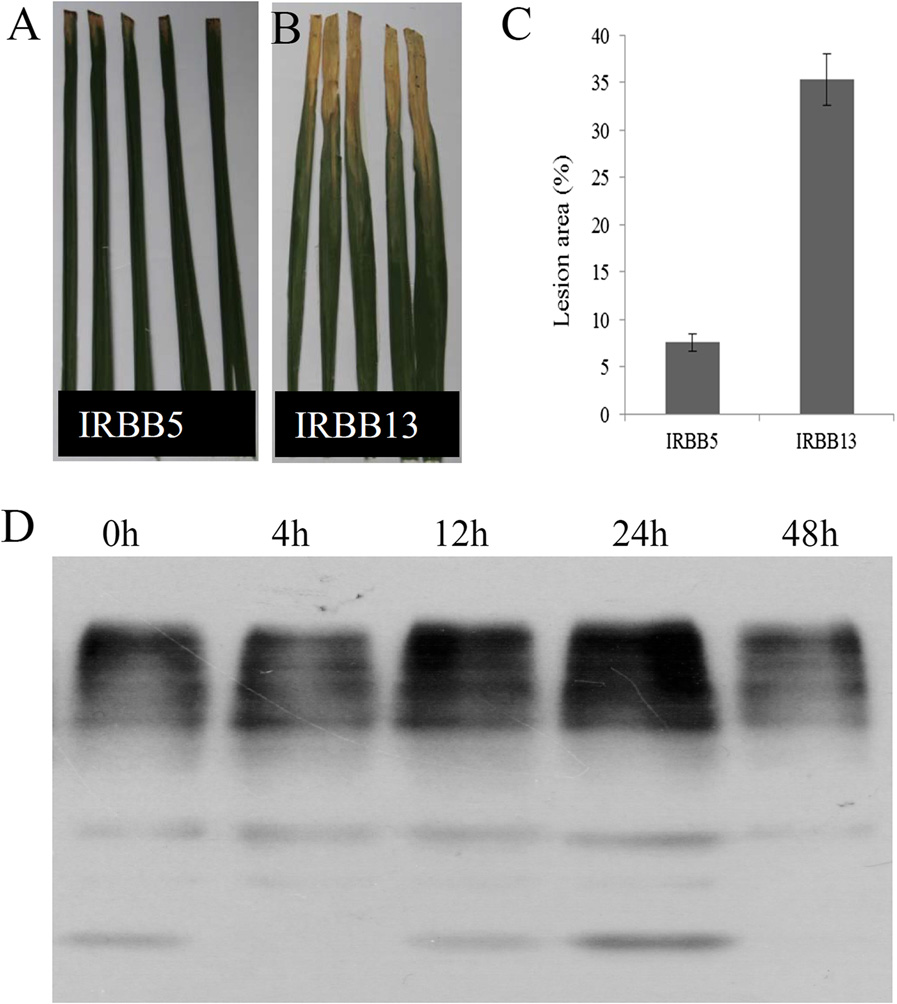
The phenotype of IRBB5 and IRBB13, and global phosphorylation dynamics of IRBB5 under *Xoo* infection. **a** and **b** The phenotype of IRBB5 and IRBB13 under *Xoo* infection, respectively. **c** The lesion area counted for IRBB5 and IRBB13. **d** Western-blot analysis of IRBB5 globe phosphorylation dynamics at the different time points under *Xoo* infection

A BB resistant variety IRBB5 was used as the starting material in this study due to its good performance against BB (Fig. 1a and b). Our infection assay found that the lesion area of IRBB5 was only around 7 % when the *Xoo* strain zhe173 was inoculated for 10 days, while IRBB13, a BB susceptible variety, showed over 35 % lesion area under the same condition (Fig. 1c), suggesting IRBB5 is highly resistant to BB. To gain a global view of the phosphorylation dynamics of IRBB5 in response to BB, Western blot analysis was conducted for the leaf total protein samples at different time points after zhe173 inoculation. For each sample, equal amount of total protein (100 μg) was loaded for the assay. As shown in Fig. 1d, multiple bands were detected in all the samples and phosphorylation signal intensity of several bands have been changed during the inoculation of *Xoo*, suggesting protein phosphorylation plays important roles in rice disease resistance. Interestingly sample collected after 24 h of inoculation showed more intense phosphorylation signal than protein samples from other time points in Western blot analysis and it also indicated the further exploration of phosphosites is worth studying from 24 h protein sample.

### Identification of phosphorylation sites, peptides and proteins

**Fig. 2:**
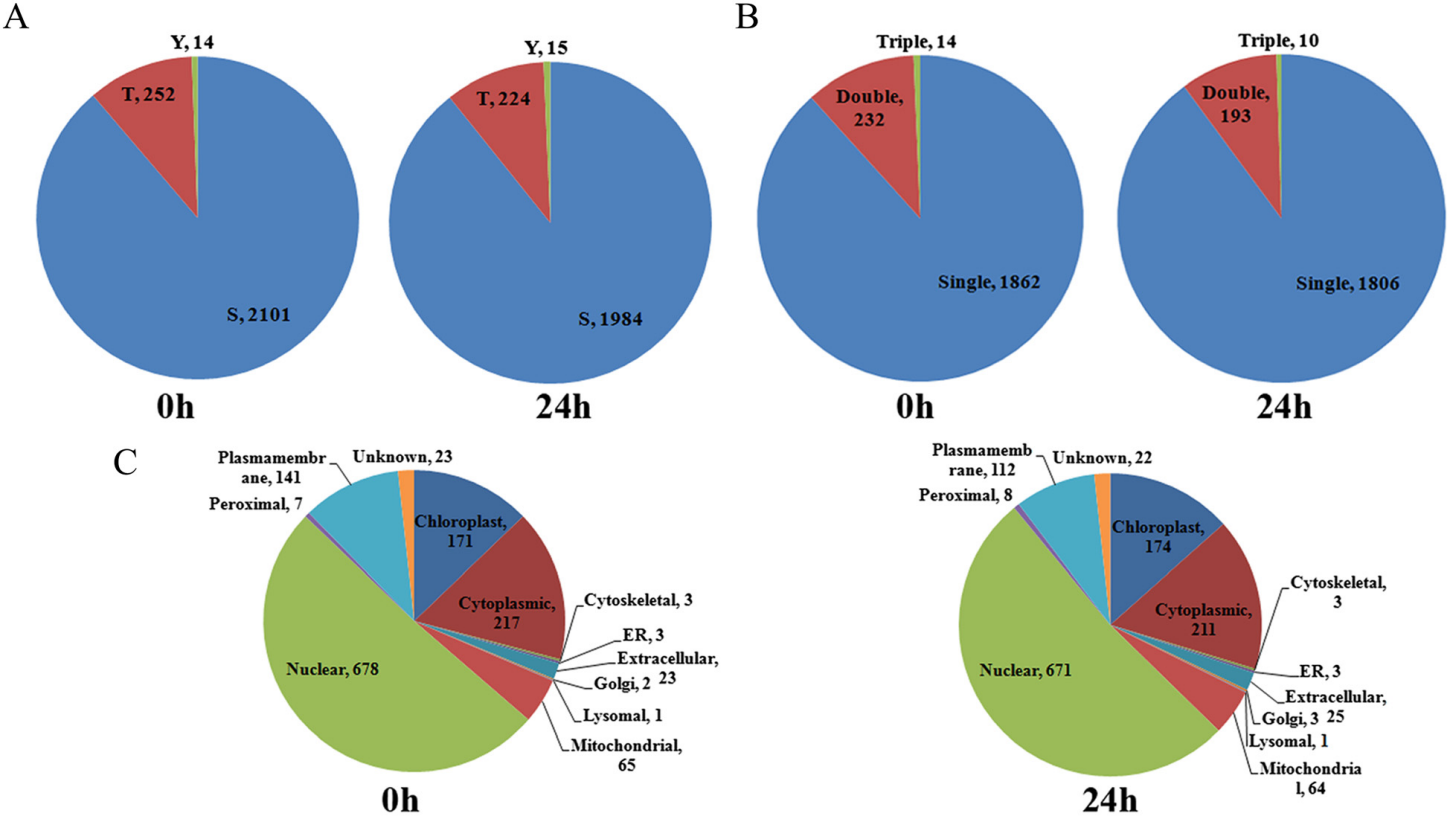
The distribution of phosphosite types and subcellular localization of phosphoproteins. **a** Pie chart showing the distribution of phosphoserine, phosphothreonine and phosphotyreosine. **b** Pie chart showing the number of phosphopeptides carrying multiple phosphosites. **c** The subcellular localization distribution of phosphoproteins

To explore the role of protein phosphorylation in rice disease signaling and resistance, a quantitative, non-gel, label-free phophoproteomic study was conducted for the leaf samples of IRBB5 at the time points of 0 h and 24 h after *Xoo* infection with three biological replicates. Phosphopeptides were enriched from leaf total proteins by TiO2-MOAC (Metal oxide affinity chromatography) method followed by LC-MS/MS assay. In the current study, a total of 2108 and 2009 phosphopeptides were identified in 0 h and 24 h samples, representing 1334 and 1297 proteins, respectively (Additional file 1: Table S1). In the 2108 phosphopeptides of 0 h, there were 2367 phosphosites, including 2101 serine (88.8 %), 252 threonine (10.6 %) and 14 tyrosine (0.6 %) sites. Similarly, in the sample of 24 h, all 2009 phosphopeptides covered 1984 serine, 224 threonine and 15 tyrosine phosphosites, representing a percentage of 89.2 %, 10.1 % and 0.7 % of the all 2223 phosphosites respectively (Fig. 2a). The distribution of phophorylation types in our study is consistent with other reports in rice, *Triticum aestivum* and *Brachypodium distachyon* [14–16]. In both 0 h and 24 h samples, most of the peptides carried only one phosphorylation modification; around 10 % peptides carried two phosphorylations, whereas three phosphorylation modifications occurred in less than 1 % of the peptides (Fig. 2b).

### Conserved phosphorylation motifs analysis of the unique phosphpeptides

**Fig. 3:**
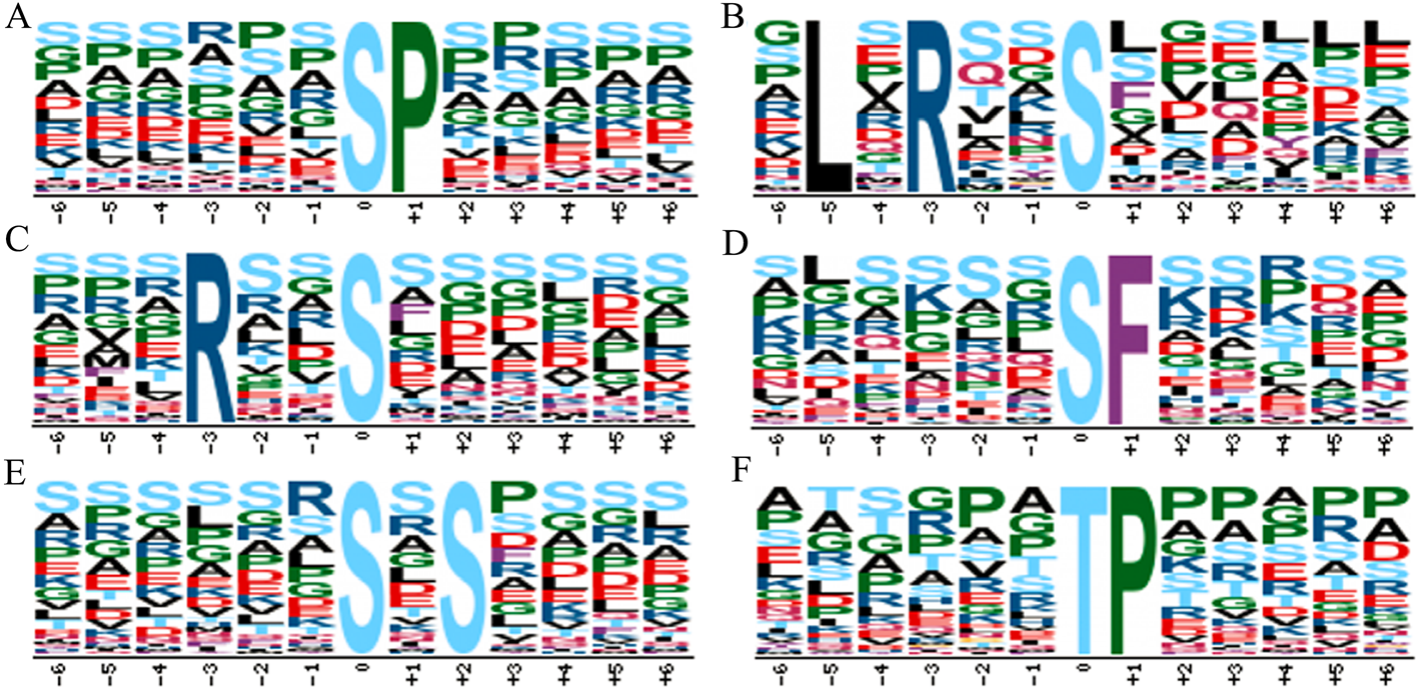
Over-presented amino acid motifs detected from the identified phosphosites by Motif-X. **a**-**e** Five enriched motifs from phosphoserine. **f** Enriched motif from phosphothreonine

By using the Motif-X tool (http://motif-x.med.harvard.edu/motif-x.html) [17], the over-presented motifs around the phosphosites were analyzed. Firstly, a 13 amino acid (AA) sequence centered by the phosphorylation site were extracted from both 0 h and 24 h phosphopeptides. After removing the redundant sequences from both datasets, we obtained totally 2303 unique amino acid sequence extractions, including 2040 centered by phsophoserine, 247 centered by phosphothreonine and 16 tyrosine-centered phosphopeptides. Due to the small number of phosphorylated tyrosine sites, no obvious conserved motif was detected in our assay. Intriguingly, at least five types of conserved motifs were significantly enriched around the phosphoserine sites (Table 1 and Fig. 3). [sP] was the most common motifs as 1214 matches were found in our result. Followed were [Rxxs] and [sxS] with over 500 hits been detected. There were also more than 100 hits of [LxRxxs] and [sF] motifs. Nevertheless, to the best of our knowledge, [sF] was not found in any other reports in plants except to this study, which possibly due to the fact that different protein extraction methods and plant tissues were used in different studies. On the other hand, the very limited phosphosite data accumulation in plants would also be a reason for this phenomenon. As for phosphothreonine, [tP] was the only conserved motif found in this study. Recent studies have revealed numerous over-presented motifs from plants, and linked them with certain kinase substrates [18]. Besides this research, [sP] motif was over-presented in other studies in *Arabidopsis*, rice and wheat [14, 18, 19]. This proline-directed motif could be a potential targets for MAPK, SnRK2 (sucrose non-fermenting1-related protein kinase 2), RLK (receptor-like kinase), AGC (cAMP-dependent, cGMP-dependent and protein kinase C), CDK (cyclin-dependent kinase), CDPK (calcium-dependent protein kinase) and SLK (STE20-like kinase) kinases [18]. [Rxxs] motif could be recognized by MAPKK, CaMK(calmodulin-dependent protein kinase)-II and protein kinase A [14, 18]. Though [sxS] has been detected by some researches, its potential kinases remain unknown yet [18]. By far, [tP] is the most common phosphothreonine motif found in plants [18].

**Table 1:** Motif-X analysis of unique phosphopeptides

Motif	Motif score	Foreground matches	Foreground size	Background matches	Background size	Fold increase
[sP]	16	1214	6054	3860	40638	2.11
[LxRxxs]	27.45	106	4840	228	36778	3.53
[Rxxs]	16	557	4734	2671	36550	1.61
[sF]	14	176	4177	760	33879	1.88
[sxS]	8.37	733	4001	4958	33119	1.22
[tP]	16	138	524	511	5036	2.6

Lower case “s” and “t” indicate phosphoserine and phosphothreonine respectively. “x” represents any amino acid

### Subcellular localization of phophoproteins

To predict the putative subcellular localization of phosphoproteins, the sequences of both 0 h and 24 h phosphoproteins were used to search against the “Eukaryotes” database of CELLO with default setting (http://cello.life.nctu.edu.tw/) [20, 21]. The results showed that all of the 0 h and 24 h phosphoproteins obtained a hit respectively, and both showed very similar cellular compartment distributions (Fig. 2c). Over 50 % of the phosphoproteins were located in the nuclus, followed by cytoplasm, chloroplast and plasmamembrane localized proteins. However, other compartments, such as mitochondrial, golgi and ER, had less than 20 % of the phosphoproteins in total. So far, no report regarding the cellular compartments distribution of rice leaf phosphoproteins is available, but several other cases in rice pistil, cotton leaf or *physcomitrella patens* protoplast showed divergent distribution patterns [16, 19, 22]. The difference in distribution patterns may be due to the differences in species, tissues or methods used for protein extraction.

### Differentially Phosphorylated (DP) peptides and proteins in response to *Xoo* infection

Based on the average phosphorylation intensity of three biological replicates, 1070 DP peptides were screened out with 2 fold change or more (P < 0.05), including 427 up-phosphorylated and 643 down-phosphorylated peptides after *Xoo* infection (Table 2). In the up-phosphorylated peptides, 342 peptides were specifically phosphorylated in 24 h, but not in 0 h, while the other 85 showed over 2 fold intensity increasing after infection. We also found 441 (68.6 %) of the down-phosphorylated peptides were specifically phosphorylated in 0 h, and the other 202 peptides had decreased intensity less than 0.5 folds. In addition to the DP peptides, there were 1380 phosphopeptides showing no significant changes in intensity after *Xoo* infection, suggesting that they may be functionally unrelated to disease resistance.

**Table 2:** Numbers of differentially phosphopeptides and phosphoproteins

	0 h specifically modified	24 h specifically modified	0 h/24 h up-phosphorylated^a^	0 h/24 h down-phosphorylated^b^	0 h total	24 h total
Phosphopeptide	441	342	85	202	2108	2009
Phosphoprotein	272	298	53	139	1147	1130

^a^The phosphorylation intensity of 0 h/24 > 2 folds, *P* < 0.05

^b^The phosphorylation intensity of 0 h/24 < 0.5 folds, *P* < 0.05

A database search of the phosphopeptides resulted in the identification of 1302 corresponding phosphoproteins, among which there were 762 DP proteins with 53 being up-phosphorylated and 139 being down-phosphorylated after *Xoo* infection (Additional file 1: Table S1). We also found that there were 272 and 298 proteins that were specifically phosphorylated in 0 h and 24 h respectively. Transcription factor (TF) is a major group of the DP proteins as 62 TFs were identified, including 38 down-phosphorylated and 24 up-phosphorylated (Additional file 2: Table S2). Furthermore, the DP proteins covered 28 epigenetic control factors whose function were involved in DNA methylation, histone methylation, chromatin condensing etc.; implying that cross-talk of various PTMs plays important roles in the plant disease resistance (Additional file 2: Table S2).

Differential phosphorylation pattern usually indicates the regulatory roles of the DP protein in the corresponding biological process. Up to date, numerous high through-put, quantitative studies have been reported investigating the phosphorylation dynamics in seed development, seed germination, fruit ripening, abiotic stress in *Arabidopsis*, *maize, rice, soybean, sweet orange* and *wheat* [14, 23–27]. Previous studies also clearly showed that phosphorylation/dephosphorylation of signaling proteins transmit messages from the pathogen secreted elicitor to the cell nucleus, where the immune reaction could be triggered upon the message reception [28, 29]. Nevertheless, few literatures describing plant phosphoproteome to biotic stress are available so far. Only five differentially phosphorylated proteins were found in *Arabidopsis* during the defense response to *Pseudomonas syringae* pv. tomato DC3000 [30]. In *grape vine*, 48 proteins were found to be differentially changed in abundance or/and phosphorylation intensity under *Flavescence dorée* phytoplasma infection [31]. Benschop et al. (2007) found 76 membrane-associated proteins including a number of defense-related proteins were differentially phosphorylated from *Arabidopsis* cells treated with bacterial elicitor flg22 or fungal elicitor xylanase [32]. Recently, in a study of the rhizobia-root hair infection process in soybean, 273 phosphopeptides corresponding to 240 phosphoproteins were found to be significantly regulated in response to inoculation with *Bradyrhizobium japonicum* [33]. The large number of DP proteins identified in this study could be valuable candidate proteins to reveal the phosphorylation-mediated plant disease resistance.

### Gene Ontology analysis of DP proteins

**Fig. 4:**
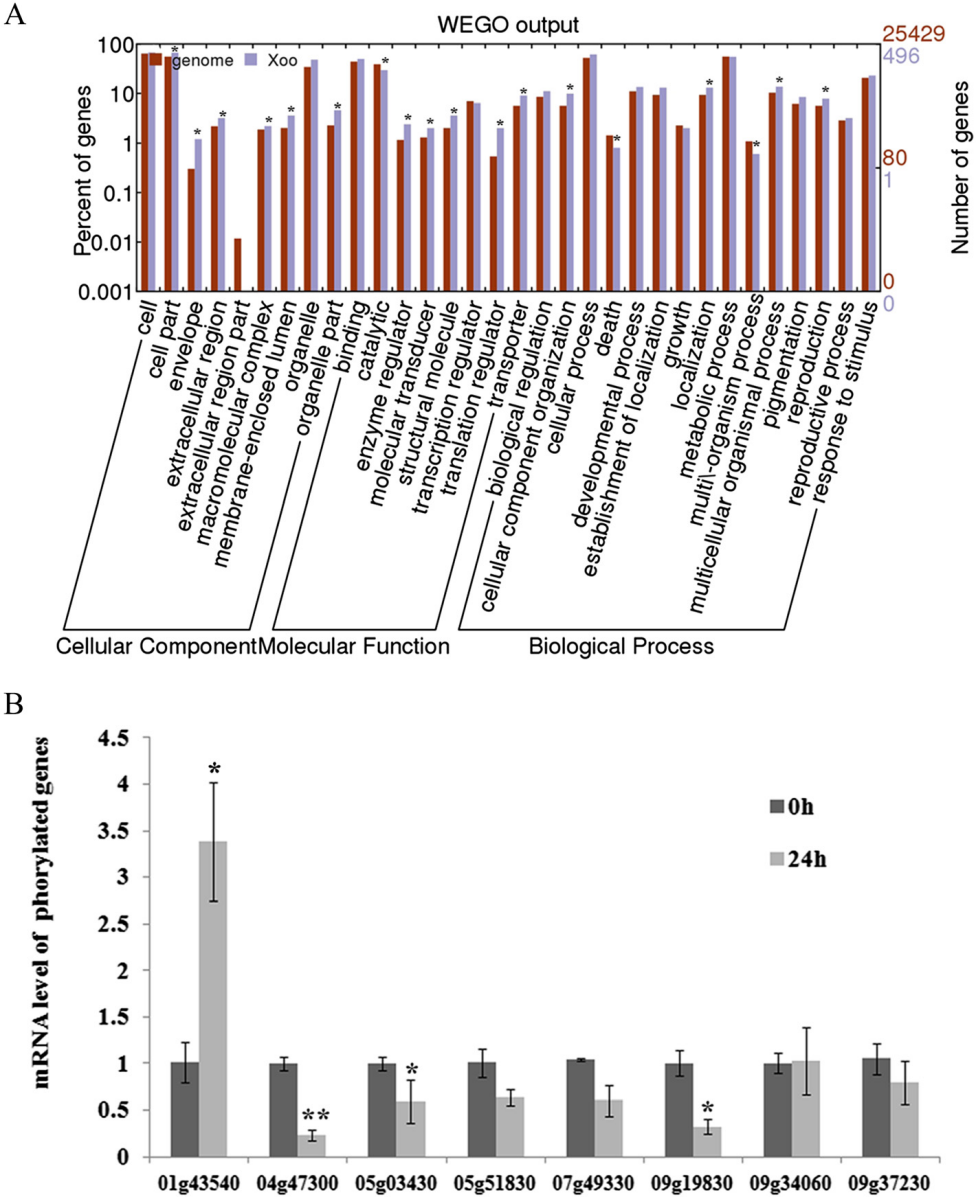
GO analysis of DP proteins (**a**) and quantitative RT-PCR analysis of the mRNA abundance of the corresponding DP protein genes (**b**). Standardized residue was employed for the statistical analysis of GO enrichment, where standardized residue [=(Observed-expected)/√expected], which follows asymptotically a normal distribution [86]. An absolute SR value larger than 2.33 indicates statistical significance at *P* < 0.01. Based on the distribution of each GO category in genome, an expected number of DP proteins in each GO category could be calculated out. Observed is the number actually occurred in each GO category * indicates *P* < 0.05; ** indicates *P* < 0.01

The agriGO online software was employed to classify DP proteins based on their gene ontology annotations in the vocabulary of “cellular component”, “biological process” and “molecular function” (Fig. 4a). From the “cellular component” perspective, envelope, cell part, macrocellular complex, membrane-enclosed lumen, organelle part and extra cellular region part were over-presented in our DP proteins when the whole-genome encoding proteins was used as a control (P < 0.05). In terms of “molecular function”, enzyme regulator, structural molecule and translation regulator were significantly enriched in DP proteins, while catalytic was less presented than the control (P < 0.05). From the perspective of “biological process”, DP proteins were preferentially cataloged into multicellular organismal process and reproduction, whereas death and multi-organism process were less preferred (P < 0.05).

### mRNA abundance of the corresponding DP proteins

Previous transcriptomic analysis has revealed that 1601 genes were differentially expressed in rice BB resistant variety IRBB21 at the time point of 24 h after *Xoo* infection [34]. In this study, the transcriptomic data was downloaded to investigate the correlation of the mRNA transcript abundance with the protein phosphorylation intensity level. Interestingly, among the 762 DP proteins, the mRNA transcript expression of 678 DP proteins remained unchanged after *Xoo* infection (P < 0.05 and FDR < 0.05) (Additional file 3: Table S3). Moreover, even for those DP proteins whose mRNA level were responsive to *Xoo* infection, no clear correlations were found between the mRNA abundance and phosphorylation intensity, indicating that the phosphorylation intensity variation detected in our study was majorly due to the occurrence of phosphorylation event in the pre-existing proteins, instead of the quantity change caused by protein synthesis or degradation. This hypothesis is also supported by our quantitative RT-PCR of 8 randomly selected DP protein genes (Fig. 4b). Our qRT-PCR results showed that the transcription expression level of four genes (LOC_Os05g51830, LOC_Os07g49330, LOC_Os09g34060 and LOC_Os09g37230) were not significantly altered (P > 0.05). For the rest four genes tested, despite their mRNA expression level being significantly changed (P < 0.05) or extremely changed (P < 0.01), we noticed that the variation in the phosphoprotein level was apparently much larger than these in the mRNA level. For example, the mRNA expression level of LOC_Os05g03430 and LOC_Os09g19830 was approximately 40 % and 70 % down-regulated by *Xoo* infection, whereas the phosphorylation was completely removed for both proteins. Taken together, the results above may suggest that the phosphorylation intensity, rather than the quantity, of the proteins essentially regulates the plant disease resistance.

#### Protein-protein interaction (PPI) analysis of DP proteins

**Fig. 5:**
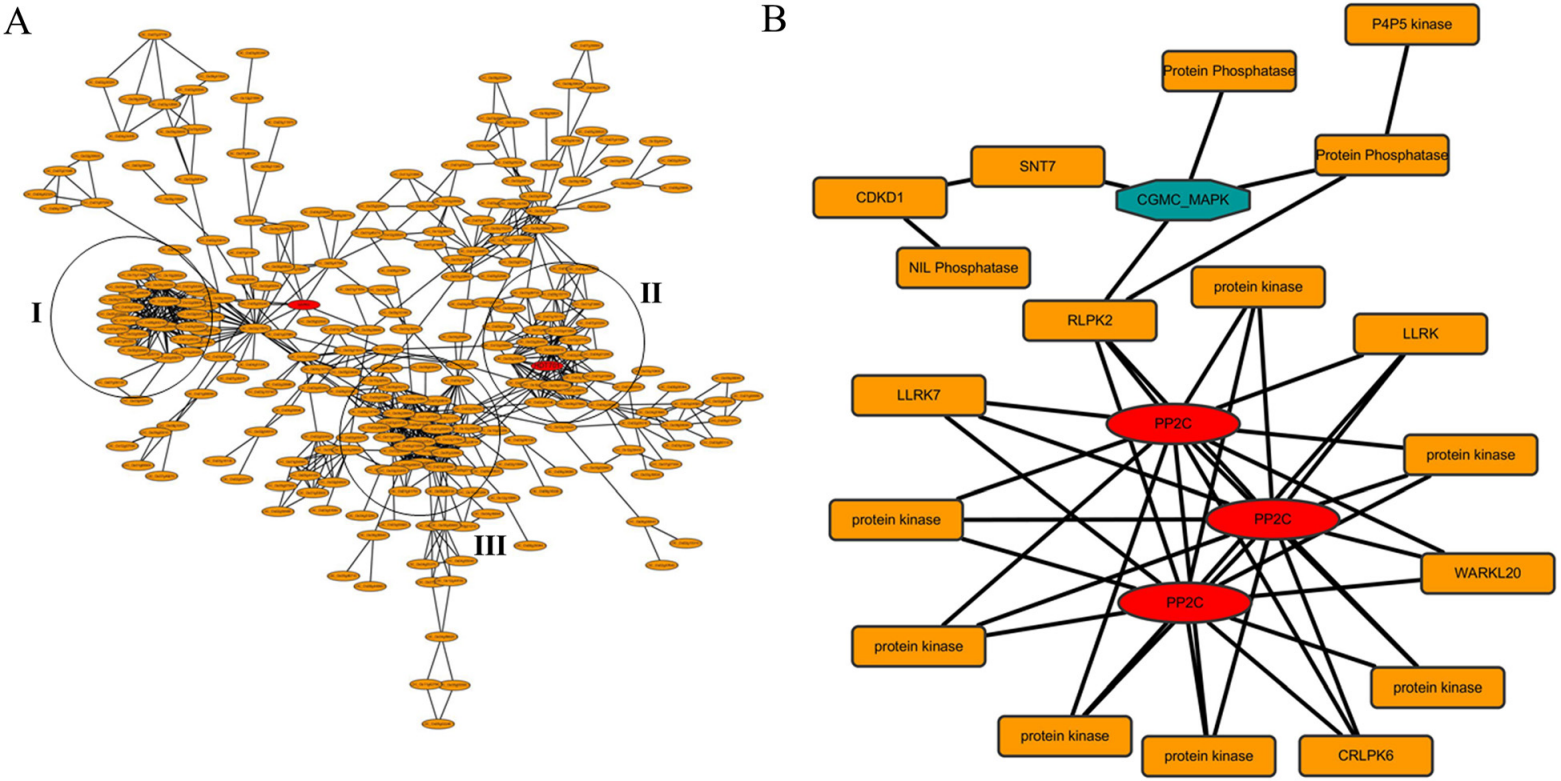
A sub-network of all the DP proteins (**a**) and DP kinases and phosphatases (**b**) by using STRING and Cytoscape. The locus ID of the abbreviations in (**b**) could be seen in Additional file 4: Table S4

STRING (Search Tool for the Retrieval of Interacting Genes/Proteins) version 10.0was employed in this study for the potential PPI analysis of the DP proteins (http://string-db.org/) [35]. The parameter for confidence score was set to 0.7 to assure a high reliability, and the yield PPI results were visualized by Cytoscape software [36]. When all the 762 DP proteins were used as input for the analysis, the yield result displayed a complicated network with 327 nodes (proteins) and 787 edges (interaction relationships) (Fig. 5a). We found three groups of DP proteins were aggregated, including HDT701 in group II, suggesting intense interactions among these interaction partners. To gain an in-depth view of the phosphorylation-mediated signaling, we also analyzed the PPI of the kinases and phosphatases of the DP proteins. As shown in Fig. 5b, a network comprising 22 nodes (Additional file 4: Table S4) and 44 edges was obtained. Interestingly, three PP2Cs were centered in the network, suggesting the ABA related signaling plays important roles in the plant disease resistance.

## Discussion

In this study, a quantitative, MS-based, label-free proteomic analysis identified 2450 non-redundant phosphopeptides from 1302 phosphoproteins of rice at both 0 h and 24 h after *Xoo* infection including 762 differentially phosphorylated proteins, representing the first phosphoproteomic attempt to explore the phosphorylation events in rice-pathogen cross-talk.

### Phosphorylation-dependent signaling

Through the phosphorylation/dephosphorylation of kinase cascade controlled by kinase or phosphatase, the signals of pathogen infection stimuli could be transmitted to the nucleus, where disease resistant-related proteins will be directly or indirectly phosphorylated or dephosphorylated to initiate the immune response. Phytohormone abscisic acid (ABA) signaling is well-known for its roles in response to abiotic stress as well as to biotic stress [37, 38]. A new “PYR/PYL/RCAR (an ABA receptor)-PP2C (type 2C protein phosphatase)-SnRK2”cascade model for ABA signaling has been proposed and validated, in which the soluble PYR/PYL/RCAR receptors function at the apex of a negative regulatory pathway to directly regulate PP2C phosphatases, which in turn directly regulate SnRK2 kinases. In rice, there are at least 78 PP2Cs have been identified [39]. Intriguingly, four PP2C proteins were found differentially phosphorylated in our result, among which OsPP2C27 (LOC_Os02g55560) and OsPP2C57 (LOC_Os06g39600) were up-phosphorylated by *Xoo* infection (Table 3). As negative regulators, PP2Cs competitively bind with ABA receptors to relieve the inhibition on SnRKs under stress conditions. The up-phosphorylation of OsPP2C27 and OsPP2C57 probably promote the binding to ABA receptors, thus to trigger the ABA-dependent signaling in rice defense. This hypothesis is supported by similar results from *wheat*, in which two PP2Cs were up-phosphorylated by drought stress [14].

**Table 3:** Some examples of the differentially phosphorylated proteins identified in this study

Peptide sequence	Protein group accessions	Annotation	Reported function	Ref	Modifications on peptide	Phos intensity 24 h/0 h
FLTASGTFKDGELR	LOC_Os01g32660.4	STE_MEK_ste7_MAP2K.2	Involved in cold stress signaling	[65, 66]	T7 (Phospho)	24 h specific
NQHLLSPR	LOC_Os01g60700.1	Serine/threonine-protein kinase NAK			S6 (Phospho)	24 h specific
RSQEEDEVEER	LOC_Os02g50970.1	Protein kinase domain containing protein	Mediates drought resistance through ROS scavenging	[67]	S2 (Phospho)	0 h specific
SISAEGLHSLR	LOC_Os02g55560.1	Protein phosphatase 2C, putative			S3 (Phospho)	24 h specific
LTSVVEEDNRGEEVVEEEAR	LOC_Os03g18070.1	Omega-3 fatty acid desaturase, chloroplast precursor	May be involved in heat tolerance	[68]	S3 (Phospho)	0 h specific
MSPAEASREENVYMAK	LOC_Os03g50290.1	14-3-3 protein	Involved in Biotic and Abiotic Stress response	[69]	N-Term (Acetyl); S2 (Phospho)	24 h specific
APSSAGAAAGRPGLMVLR	LOC_Os03g59390.1	CAMK_CAMK_like.24			S3 (Phospho)	2.147117423
LMDYKDTHVTTAVR	LOC_Os04g38480.1	BRASSINOSTEROID INSENSITIVE 1-associated receptor kinase 1 precursor	Regulates rice leaf development	[70]	T7 (Phospho); T10 (Phospho)	24 h specific
NAPGGPLSPGGFPMNRPGTGGMMPGMPGTPGMPGSR	LOC_Os04g42140.1	Eukaryotic initiation factor iso-4 F subunit p82-34	Confers high resistance of rice to Rice yellow mottle virus	[43, 71]	S8 (Phospho)	0 h specific
ASGGGGEMGPVLQR	LOC_Os04g47300.1	CAMK_CAMK_like.26	Oppositely modulates salt-stress tolerance and blast disease resistance	[40]	S2 (Phospho)	0.4237053
HDTDDNNNAAAADSPKKPSRPPAAAK	LOC_Os04g49510.1	CAMK_CAMK_like.27	Confers both cold and salt/drought tolerance on rice	[72]	S14 (Phospho)	0 h specific
EMSDDESTDKLLVEPQK	LOC_Os04g58620.1	Potasium efflux antiporter protein	Regulates chloroplast development and drought resistance	[73]	S3 (Phospho)	0.290186904
ALNNIMHMSNSPTSSYR	LOC_Os05g03430.3	ATSIZ1/SIZ1, E3 Ubiquitin ligase	Regulates Vegetative and reproductive Development, enhances broad abiotic stress tolerance	[74]	T13 (Phospho)	0 h specific
SIHGSQLGTVTEAEHS	LOC_Os05g05590.1	Transporter, monovalent cation:proton antiporter-2 family	Enhances rice sanity tolerance	[75]	S1 (Phospho)	0.496889646
KLVNSSFADLQKPQMELDGK	LOC_Os05g38150.1	Amino acid synthetase	Enhances rice sanity and drought tolerance	[76]	S5 (Phospho)	0 h specific
TINESMDELSSQSK	LOC_Os05g47560.1	Serine/threonine-protein kinase SNT7, chloroplast precursor			T1 (Phospho); M6 (Oxidation)	24 h specific
IAHIPKPEASLDSLSFK	LOC_Os05g50710.1	Late embryogenesis abundant protein	Enhances the cell tolerance to various biotic and abiotic stresses	[77]	S15 (Phospho)	24 h specific
VSQPAEEDEMDFDSEEVEDEEEEEK	LOC_Os05g51830.1	ZOS5-12 - C2H2 zinc finger protein, Histone Deacetylase	Negatively Regulates Plant Innate Immunity	[61]	S14 (Phospho)	0 h specific
TTSETDFMTEYVVTR	LOC_Os06g06090.2	CGMC_MAPKCMGC_2_ERK.12	Activates rice innate immunity	[41, 78]	T9 (Phospho); Y11 (Phospho)	0.473270364
LYEHGATPATTR	LOC_Os06g10790.1	Lectin-like receptor kinase			T10 (Phospho)	24 h specific
DFGSMNMDELLR	LOC_Os06g10880.3	bZIP transcription factor	Responds to ABA and IAA	[79]	S4 (Phospho)	24 h specific
QIDASDLPSDDSADNDYDPTLAQGHK	LOC_Os06g12400.1	Homeobox domain containing protein	Regulates GA response	[80]	S5 (Phospho); S9 (Phospho)	0 h specific
SISAEGLR	LOC_Os06g39600.2	Protein phosphatase 2C, putative			S3 (Phospho)	2.320807642
DGGAASEYLIEEEEGLNEHNVVEK	LOC_Os06g43660.3	Inorganic H+ pyrophosphatase	Enhances rice chill tolerance	[81]	S6 (Phospho)	0 h specific
NVSPAEQSAADK	LOC_Os06g44210.1	Protein phosphatase 2C, putative			S3 (Phospho)	0 h specific
RPFPPPSPAK	LOC_Os06g50030.1	CAMK_CAMK_like.30			S7 (Phospho)	3.145113364
SFDELSDDEGLYEDSD	LOC_Os07g39870.2	Eukaryotic peptide chain release factor subunit 1-1	Involved in chill and drought stress	[82]	S6 (Phospho)	0 h specific
ITVLTSDGSTARPKPIQK	LOC_Os08g01900.1	E3 ubiqutin ligase	Reduced cellular oxidative stress	[48]	S9 (Phospho)	0.419643669
LNSFYISHNR	LOC_Os08g14950.1	Receptor-like protein kinase 2 precursor			S3 (Phospho)	24 h specific
AGAGAGASPGWPQR	LOC_Os08g39100.1	Protein phosphatase 2C, putative			S8 (Phospho)	0 h specific
AEELVGASPGTEGMSSAEAK	LOC_Os09g34060.1	Transcription factor RF2a	Enhances rice resistance to rice tungro disease	[58, 59, 83]	S8 (Phospho)	24 h specific
SSTPAAAAEQEHR	LOC_Os10g42430.1	Transcription factor MYC7E	Involved in JA signaling	[44]	S1 (Phospho)	24 h specific
SPHGGDGDGAAGDDGGDAQAAAAGGR	LOC_Os11g29870.1	OsWRKY72 - Superfamily of TFs having WRKY and zinc finger domains	Involved in ABA response	[84]	S1 (Phospho)	24 h specific
GSLGSLNMITGK	LOC_Os12g13170.3	Transcription factor	Involved in ABA response	[85]	S5 (Phospho)	24 h specific

CDPKs are directly activated by the binding of Ca^2+^ to the calmodulin-like domain, and activated CDPKs regulate downstream components of calcium signaling. In our result, totally 8 CDPKs were identified with 6 being up-phosphorylated (Table 3). OsCPK12 (LOC_Os04g47300) is one of the documented down-phosphorylated CDPKs in our result. Literature showed that *OsCPK12*-OX seedlings had increased sensitivity to abscisic acid (ABA) and increased susceptibility to blast fungus, probably resulting from the repression of ROS production and/or the involvement of OsCPK12 in the ABA signaling pathway [40]. The differential phosphorylation pattern of OsCPK12 upon *Xoo* infection suggested that it is involved in response to multiple pathogen attacks besides blast fungus. Moreover, in agreement with the previous report, dephosphorylation of OsCPK12 detected in our data probably resulted in an “inactive” status of this negative regulator to eliminate its inhibition effect, thus enhance plant resistance to pathogen attack. In addition to PP2Cs and OsCPK12, we totally identified over 80 differentially phosphorylated kinases or phosphatases, like LRR transmembrane protein kinase (LOC_Os03g03570), MAP2K (LOC_Os01g32660) etc., suggesting that the signaling of rice-*Xoo* interaction is a very complex event with multiple signaling pathways involvement.

### Rice disease resistant-related proteins

Among the 762 DP proteins detected in this study, several proteins are functionally related to rice disease resistance (Table 3). For example, OsMAPK6 (LOC_Os06g06090), a key component in the OsRac1-OsMAPK3/6-RAI1-PAL1/OsWRKY19 rice immunity signaling cascade, was down-phosphorylated at 24 h. Previous studies have revealed that OsRac1 is a key regulator involved in basal resistance by inducing the ROS production or suppressing the ROS scavenging. OsRac1 could physically bind to OsMAPK6 and post-translationally activate OsMAPK6. Meanwhile, OsMAPK6 could directly phosphorylate RAI1, a putative basic helix–loop–helix transcription factor, the overexpression of which substantially enhanced the rice resistance to blast fungus, probably via regulating PAL1 and OsWRKY19 [41]. Though it has been clear that OsMAPK6 acts as a carrier transmitting the phosphorylation from OsRac1to RAI1 in this defense signaling cascade, how OsMAPK6 is phosphorylated remains unknown. Our phosphoproteomic data indicated that the Threonine 225 and tyrosine 227 are two potential phosphosites in OsMAPK6, which will be further confirmed by our future study. We also noticed that OsMAPK6 was down-phosphorylated at 24 h, although OsMAPK6 is reported to be a positive regulator in the plant immunity response. Lieberherr et al. found that the mRNA expression of *OsMAPK6* started to decrease at 24 h after sphingolipid elicitor treatment, indicating that OsMAPK6 may be involved in the early response to pathogen infection [42]. Checking of the phosphorylation intensity of OsMAPK6 at an earlier time point, like 2 or 4 h after *Xoo* infection may be necessary to explore its functions in the future.

Another differentially phosphorylated protein gene example is *rice yellow mottle virus resistance 1*(*rymv1*, LOC_Os04g42140), a recessive gene controlling rice resistance to rice yellow mottle virus. According to our data, RYMV1 was dephosphorylated in response to the *Xoo* infection, suggesting that RYMV1 may play a negative role in bacterial disease resistance. Albar et al. (2006) cloned this gene from rice variety Giganta through a map-based strategy, and found *rymv1* is an isoform of the eukaryotic translation initiation factor 4G (eIF(iso)4G). Compared with susceptible varieties, resistant varieties present specific alleles, characterized by either amino acid substitutions or short amino-acid deletions in the middle domain of the protein [43]. Our evidences indicated that RYMV1 might be subject to the activation of phosphorylation upon the *Xoo* infection. However, whether *rymv1* mediates resistance to rice bacterial blight or not needs to be further studied by genetic analysis and pathogen inoculation assay.

In addition to OsSGT1 and RYMV1, differential phosphorylation occurred on many other potential disease resistance-related proteins, like OsMYC2 (LOC_Os10g42430) which is involved in the jasmonic acid mediated signaling [44], OsCATC (LOC_Os03g03910) and PUB15 (LOC_Os08g01900) which mediate the process of H2O2-induced cell death in rice [45–48]. Furthermore, some abiotic stress related proteins, such as OsAPX (LOC_Os03g17690) and OsHSP74.8 (LOC_Os09g29840) were also differentially phosphorylated (Table 3). These proteins have proven to be involved in rice drought, cold or heat resistance, but their biological function in biotic stress remains to be explored [49, 50].

### Transcription factors

Transcription factors (TF) serve as important internodes in the disease resistant pathway by linking the MAPKs signal with the downstream transcriptional reprogramming. Overexpression of OsBWMK1, a rice MAPK kinase, conferred plants enhanced pathogen resistance via directly phosphorylating transcription factors OsEREBP1 and OsWRKY33 [51, 52]. Similar cases were reported in other plant species as well [53–55]. In our study, at least 49 transcription factors were differentially phosphorylated, including 36 down-phosphorylated and 13 up-phosphorylated, according to the rice transcription factor list released from DRTF database (Database of Rice Transcription Factors, http://drtf.cbi.pku.edu.cn/) [56]. These TFs consist of bZIP, bHLH, Myb and WRKY family members. Intriguingly, RF2a (LOC_Os09g34060), a bZIP domain containing TF was specifically phosphorylated after *Xoo* infecton. Dai et al. (2008) found that overexpression of RF2a could repress the symptoms of rice tungro disease without having any growth penalty. They suggested that RF2a together with RF2b suppress the RTBV replication via directly binding to its *cis* element box II in the promoter [57–59]. As indicated in our qRT-PCR results, *Xoo* infection did not alter the mRNA abundance of RF2a, but changed the protein status from unphosphorylated to phosphorylated, implying that RF2a plays essential roles in bacterial blight resistance, and phosphorylation might be an important step for the activation of pre-existing RF2a. In addition to RF2a, many other TFs such as WRKY, Myb and bHLH were found to be differentially phosphorylated, which suggested that they are good candidate genes for rice bacterial blight resistance.

### Epi-genetic controlling factors

Recent studies demonstrated that chromatin remodeling accomplished through histone modifications is emerging as a key process in the orchestration of plant biotic stress responses and epi-genetic controlling factors are the critical regulators of plant defense to pathogen attack. Not surprisingly, many epi-genetic controlling factors were differentially phosphorylated in this study. HDT701/OsHDT1encoding a histone deacetylase plays versatile roles in plant development and stress response. Li et al. (2011) reported that *HDT701/OsHDT1*expression displayed a circadian rhythm. Elevated *OsHDT1* expression imposed no effects on plant growth in the parent but led to early flowering in the hybrid. It was suggested that HDT701/OsHDT1may be involved in epigenetic control of parental genome interaction for differential gene expression [60]. Besides the control of hybrid flowering, its role in rice innate immunity was unraveled in a recent publication [61]. Transcription of *HDT701* could be induced by compatible reaction and repressed by the incompatible reaction after infection by the fungal pathogen *Magnaporthe oryzae* (*M. oryzae*). More importantly, in the rice *HDT701* overexpression lines, the global histone H4 acetylation level was reduced and plants became more susceptible to the rice pathogens *M. oryzae* and *Xoo*. Silencing of *HDT701* imposed an opposite effect on rice in that the resistance to both *M. oryzae* and *Xoo* was enhanced. The underlying mechanism could be that HDT701 physically bind to and modulate the levels of histone H4 acetylation of pattern recognition receptor (PRR) and defense-related genes, such as *MAPK6* and *WRKY53*. In our study, we also found that HDT701/OsHDT1in a phosphorylated status at 0 h, but the phosphorylation was found to be removed by the *Xoo* infection. Given that phosphorylation modification usually activates protein function, the dephosphorylation of HDT701/OsHDT1 upon *Xoo* infection suggested that it is a negative regulator of plant defense, which is highly consistent with the conclusion of Ding et al. [61]. However, even though the mask of the biological function of HDT701/OsHDT1has been unraveled, how HDT701/OsHDT1 itself is regulated remain unclear. Our data provide good hints that phosphorylation/dephosphorylation might be a key switch turning on/off HDT701in response to *Xoo* infection. Moreover, this kind of phosphorylation switch overriding the epi-genetic regulation may be a very universal model in the plant disease resistance pathway. In this study, we have identified 17 differentially phosphorylated epi-genetic factor proteins, comprising AGO (Argonaute gene family), SNF2 chromatin remodeling complex proteins, histone demethylases, SET domain proteins etc., which are functionally related to DNA methylation, histone methylation, acetylation and ubiquitination.

## Conclusion

In conclusion, 2367 and 2223 phosphosites on 1334 and 1297 representative proteins were identified in 0 h and 24 h after *Xoo* infection, respectively. 762 proteins were differentially phosphorylated in response to the *Xoo* infection, including several well-known rice disease resistance related proteins. Our data also suggested that phosphorylation/dephosphorylation might be a key switch turning on/off many epi-genetic controlling factors in plant disease resistance pathway. To the best of our knowledge, this is the first report exploring the cross-talk of Rice-Pathogen from a view of quantitative phosphoproteome. The data obtained in this research will not only provide phosphorylation status and sites information for rice proteins, but also shed new light in studying the roles of phosphorylation in plant disease resistance.

## Methods

### Plant materials and growth conditions

Rice plants of IRBB5 (*xa5*) and IRBB13 (*xa13*) were obtained from National Rice Research Institute (CNRRI). IRBB5 (*xa5*) and IRBB13 (*xa13*) were two near-isogenic rice lines with a single gene used to characterize virulence of *Xanthomonas oryzae pv. oryzae* (*Xoo*) isolates in China. IRBB5 (*xa5*) and IRBB13 (*xa13*) seedlings were grown in the net house of CNRRI. The cultivation and management of the rice in the net house proceeded as usual.

### Rice bacterial blight inoculation

IRBB5 and IRBB13 plants were inoculated with the Chinese representative strain of *Xoo* (Zhe173) at the booting stage by the leaf clipping method [62]. The concentrations of *Xoo* suspension is up to 3x10^8^ cfu/mL. Disease was scored (3 to 5 leaves for each plant) as the percent lesion area (lesion length/leaf length) at ten days after inoculation.

### Total protein extraction

After inoculation, around 5 cm long IRBB5 leaves close to the clip position were collected immediately after *Xoo* inoculation (0 h) and at 24 h after inoculation (24 h). The total proteins were extracted using the urea-extraction method. Three individual biological replicates were used for each time point. Briefly, 1 gram of rice leaf tissue was grinded into fine powder, lysed with 5 mL lysis buffer (150 mM Tris pH8.0, 8 M urea, 1X phosphoprotein protease inhibitor complex, and 1 mM phenylmethylsulfonyl fluoride) by shaken for 30 min at 4 °C, and sheared by sonication (80 W in power, sonicate 10 s, stop 15 s to cool down, repeat 10 times). After centrifugation at 10,000 rpm for 15 min, the supernatant was aliquoted, and the proteins were precipitated in 100 % acetone, washed in 75 % ethanol and resolved in the lysis buffer. Lastly, the extracted total proteins were quantified with Bradford assay.

### Western blot analysis

The time-course phosphoprotein differences of IRBB5 inoculated *Xoo* were analyzed by Western blot using biotinylated Phos-tag^™^ zinc (II) complex (Wako). Firstly, the extracted total proteins were resolved on 10 % SDS-polyacrylamide gels, and subsequently transferred onto polyvinylidene fluoride fluoropolymer (PVDF) membrane using an electrophoretic blotting system (Bio-Rad). Then, the 500 μL solution which contains 10 μL Phos-tag^™^ BTL111, 20 μL 10 mmol/L Zn(NO3)_2_ and 1 μL streptavidin-conjugated horseradish peroxidase is centrifuged for 20 min (13,000 rpm) in a centrifugal filter device cup (NMWL = 30,000, NanosepTM 30 K, Pall Life Sciences). The rest solution is incubated with PVDF membrane in 30 mL TBST buffer (10 mM Tris, 100 mM NaCl and 0.1 % tween-20) for 1 h at room temperature. Lastly, the complexes on the membrane were detected by enhanced chemiluminescence (Pierce) method.

### Protein digestion

Protein were first reduced with 5 mM DTT in 56 °C for 30 min, then cold to room temperature, and alkylated with 20 mM IAA in dark for 30 min, at last added 5 mM DTT in dark for 15 min. The reduced and alkylated proteins were digested on the 30 kDa filter unit (Millipore) over night with trypsin at pH8.0 (with an enzyme to protein ratio of 1:50). Peptides obtained by filter-aided sample preparation (FASP) were desalted using C18 Sep-Pak (Waters).

### Phosphopeptide enrichment

The digested peptides were resolved with binding buffer (80 % ACN, 5 % TFA, 1 M lac acid), then incubated with TiO2 beads (GL sciences, peptide to TiO2 ratio of 1:4) for three times, each time for 30 min then washed with binding buffer for twice. Transfer all TiO2 beads into a 200 mL homemade StageTip that with two pieces of C18 solid phase extraction disk (3 M), phosphopeptides were washed by elution buffer (40 % ACN, 15 % NH3H2O) for 4 times. Eluates were subsequently dried to ~5ul in a SpeedVac and reconstituted with 5 % MeOH in 1 % TFA solution for LC-MS/MS analysis.

### LC-MS/MS and data analysis

Peptides were separated by using a homemade reversed-phase column (75umID x 15CM) and eluted in a 1 h 5-30 % acetonitrile gradient with an Easy-nLC1000 liquid chromatography system (Thermo), analyzed by Q Exactive Plus (Thermo). Spectral data were then searched against rice database in Proteome Discoverer 1.3 suites with Mascot software. The rice database downloaded from the website (ftp://ftp.plantbiology.msu.edu/pub/data/Eukaryotic_Projects/o_sativa/annotation_dbs/pseudomolecules/version_7.0/all.dir/). The mass tolerance was set to be 20 ppm for precursor, and it was set 50mmu for the tolerance of product ions. Oxidation (M), Acetyl (Protein-N term), and Phospho (S/T/Y) was chosen as variable modifications, Carbamidomethyl (C) as fixed modification, and one missed cleavage on trypsin was allowed. To screen out the reliable phosphopeptides, FDR (False discovery rates) were assessed using the Percolator tool within the Protein Discoverer package. The results were filtered for peptide rank 1 and high identification confidence, corresponding to 1 % false discovery rate. Low-scoring peptides (Mascot score ≤20) were excluded from the analysis when they were not further supported by additional high-scoring identifications in other replicates or experiments. For reliable phosphorylation site analysis, all phosphopeptide hits were automatically re-analyzed by the phosphoRS software within the Protein Discoverer software suite. PhosphoRS probability higher than 90 % was required for a phosphorylation site to be considered as localized. Only those peptides which were phosphorylated in at least two of the three biological replicates were considered as truly phosphorylated. The differentially phosphorylated protein was defined to have over two fold changes in the normalized average intensity with credible student’s *t*-test (P < 0.05).

### Quantitative RT-PCR (qRT-PCR)

Total RNA of IRBB5 leaves at 24 h after inoculation was isolated using Trizol (Invitrogen) according to the manufacturer’s manual. Two micrograms of total RNA was performed for reverse transcription using first strand cDNA synthesis Kit (Toyobo). For real-time quantitative RT-PCR, all the primers used are listed in Additional file 5: Table S5, and ubiquitin gene was used as an internal control. Quantitative PCR was performed in a total reaction volume of 20 microliter (10 μl THUNDERBIRD SYBR® qPCR Mix (Toyobo), 1 μl cDNA, 1 μl primers, and 8 μl water) on the LightCycler 4.80 real-time PCR detection system (Roche). Expression was assessed by evaluating threshold cycle (CT) values. The relative expression level was calculated by the 2^-ΔΔCT^ method [63]. The experiment was performed in three replicates.

### Availability of supporting data

The mass spectrometry proteomics data have been deposited to the ProteomeXchange Consortium [64] via the PRIDE partner repository with the dataset identifier PXD002222.

## **Additional files**

Additional file 1: Table S1. All phosphopeptides and DP peptides identified in this study. Additional file 2: Table S2. DP transcription factors and epi-genetic controlling 2 factors. Additional file 3: Table S3. The comparison of mRNA abundance and phosphorylation intensity changes of DP proteins in response to *Xoo* infection. Additional file 4: Table S4. The locus IDs of proteins used for the interaction network construction of DP kinases and phosphatases. Additional file 5: Table S5. Sequences of the primers used in this study.

## Abbreviations

BB: Bacterial blight
*Xoo*: *Xanthomonas oryzae* pv. *oryzae*
PTM: Post-translational modification
RLKs: Receptor-like kinases
MAPK: Mitogen-activated protein kinase
MAPKK: Mitogen-activated protein kinase kinase
MAPKKK: Mitogen-activated protein kinase kinase kinase
AGC: cAMP-dependent, cGMP-dependent and protein kinase C
CDK: Cyclin-dependent kinase
CDPK: Calcium-dependent protein kinase
SLK: STE20-like kinase
SnRK2: Sucrose non-fermenting1-related protein kinase
PP2C: Type 2C protein phosphatase
DP: Differentially phosphorylated
PRR: Pattern recognition receptor
